# Analysis of the Photophysical Behavior and Rotational-Relaxation Dynamics of Coumarin 6 in Nonionic Micellar Environments: The Effect of Temperature

**DOI:** 10.3390/molecules201019343

**Published:** 2015-10-23

**Authors:** Cristóbal Carnero Ruiz, José Manuel Hierrezuelo, José Antonio Molina-Bolivar

**Affiliations:** Department of Applied Physics II, Engineering School, University of Malaga, Malaga 29071, Spain; E-Mails: jose.hierrezuelo80@gmail.com (J.M.H.); jmb@uma.es (J.A.M.-B.)

**Keywords:** Coumarin 6, *n*-dodecyl-β-d-maltoside, Triton X-100, *n*-dodecyl-hexaethylene-glycol, nonionic micellar assemblies, time-resolved fluorescence anisotropy

## Abstract

The photodynamics of Coumarin 6 have been investigated in three nonionic micellar assemblies, *i.e.*, *n*-dodecyl-β-d-maltoside (β-C_12_G_2_), *p-tert-*octyl-phenoxy polyethylene (9.5) ether (Triton X-100 or TX100) and *n*-dodecyl-hexaethylene-glycol (C_12_E_6_), to assess their potential use as encapsulation vehicles for hydrophobic drugs. To evaluate the effect of the micellar size and hydration, the study used a broad temperature range (293.15–323.15 K). The data presented here include steady-state absorption and emission spectra of the probe, dynamic light scattering, together with fluorescence lifetimes and both steady-state, as well as time-resolved fluorescence anisotropies. The time-resolved fluorescence anisotropy data were analyzed on the basis of the well-established two-step model. Our data reveal that the molecular probe in all of the cases is solubilized in the hydration layer of micelles, where it would sense a relatively polar environment. However, the probe was found to undergo a slower rotational reorientation when solubilized in the alkylpolyglycoside surfactant, as a result of a more compact microenvironment around the probe. The behavior of the parameters of the reorientation dynamics with temperature was analyzed on the basis of both micellar hydration and the head-group flexibility of the surfactants.

## 1. Introduction

Surfactant-based systems are used in numerous applications of different technological fields, including detergency, dispersion stabilization, preparation of home and personal care products or for the increased solubility of hydrophobic materials [1,2,3]. The main feature in these applications is that these systems can, at suitable concentrations and temperatures, self-assemble in aqueous environments to form micellar assemblies. Micelles are stable nanometer-sized structures capable of solubilizing hydrophobic molecules to form “host-guest” complexes whose properties are crucial in applications such as targeted drug delivery, in the development of energy-storage devices and in different biomimetic systems [4]. Polymeric micelles formed by amphiphilic copolymers have shown a number of attractive properties to be used for applications in drug delivery [5,6,7,8]. However, due to their ability to incorporate hydrophobic molecules within their structure, micelles constituted by conventional surfactants, which are nontoxic, biocompatible and biodegradable, are also being considered as drug vehicles [9,10,11,12,13,14]. Therefore, micelles are currently emerging as nano-therapeutic agents in the pharmaceutical industry, because they can provide a useful model system in the study of fundamental interactions between the carrier, the drug and the cell, the results of which can subsequently be transferred to more complex systems.

Because of their greater tolerance to pH changes and the presence of electrolytes, better performance in the protein stabilization and reduced toxicity, nonionic surfactants are often preferred in many biochemical and pharmaceutical applications [15]. Among these materials, conventional nonionic ethoxylated surfactants have been by far the most frequently used. However, alkylpolyglycoside (APG) surfactants, characterized mainly by having one or more glucose molecules in their polar moiety, are attracting increasing interest due to their advantages in terms of biodegradability, consumer health, low toxicity and performance compared to nonionic ethoxylated surfactants [16,17,18,19]. In addition, due to their mildness and high solubilizing power, APG surfactants are being considered as possible alternatives to conventional nonionic surfactants in applications in the membrane protein field [20,21] and drug delivery [22,23].

**Figure 1:**
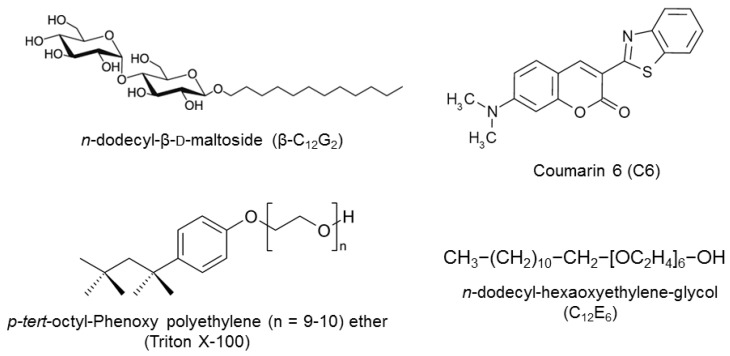
Molecular structures of the surfactants and fluorescence probe molecule used in the present investigation.

For the above reasons, our group seeks to elucidate the properties of APG surfactants as compared to those of conventional nonionic ethoxylated surfactants. In particular, we have worked to characterize mixed surfactant systems constituted by the combination of conventional ethoxylated and APG surfactants [24,25,26,27,28]. In the present paper, we have examined the effect of temperature on the microenvironmental properties of three different nonionic surfactants, two typical ethoxylated surfactants, *p-tert-*octyl-phenoxy polyethylene (9.5) ether (Triton X-100 or TX100) and *n*-dodecyl-hexaethylene-glycol (C_12_E_6_), and a representative APG surfactant, *n*-dodecyl-β-d-maltoside (β-C_12_G_2_). Figure 1 shows the molecular structures of these surfactants, indicating that the main difference between these surfactants refers to the head-group structure, which is flexible and polymer-like in the case of the ethoxylated surfactants, but bulky and rigid for maltoside. These differences involve different hydration behavior and packing conformations in the local structure of the micellar palisade layer, resulting in different structural changes upon variations in temperature [27,28].

To examine the way in which temperature affects the micellar microstructure, we studied the photophysics and dynamics of a hydrophobic dye, such as Coumarin 6 (C6) solubilized in the micellar pseudophase. Coumarin dyes have attracted much research interest in a number of areas owing to their wide applicability, and they probably constitute one of the most investigated families of probes used in microheterogeneous environments [29]. C6 (Figure 1) is a neutral probe, essentially insoluble in water, whose spectroscopic properties in homogeneous and different nano-confined systems have been previously reported [30,31,32,33,34,35]. These investigations suggest that C6 is not subject to specific interactions with its microenvironment, and therefore, local viscosity is the main factor controlling the rotational diffusion of the probe.

In this paper, we study the influence of the micellar size and hydration on the microenvironment around the probe by analyzing the effect of temperature on the photophysics and rotational diffusion of C6 solubilized in three different nonionic micelles, which were selected on the basis of their different structural response to temperature changes. In this way, using C6 as a fluorescence model drug, we aim to assess the properties of these micellar systems as encapsulation vehicles for hydrophobic drugs within a temperature range. The results will be helpful for understanding the dynamic features of other hydrophobic drugs solubilized in different micellar assemblies.

## 2. Results and Discussion

### 2.1. Spectroscopic Properties

**Figure 2:**
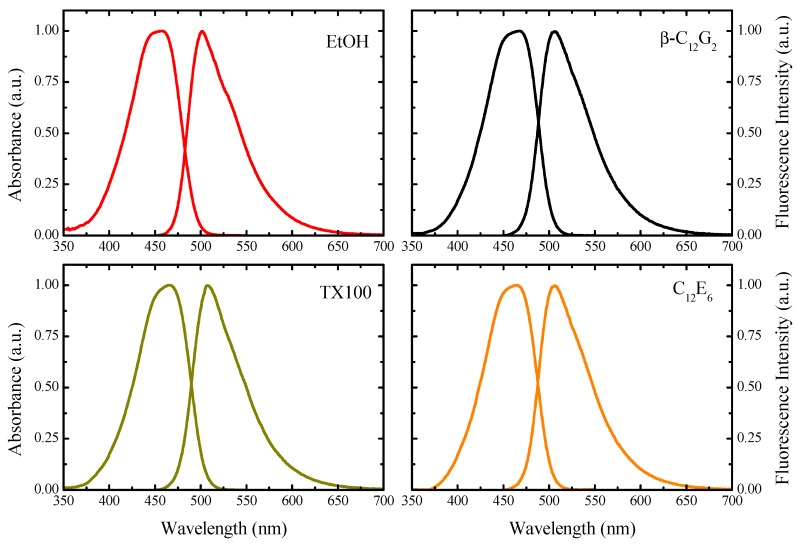
Absorption and steady-state emission spectra of C6 in ethanol (EtOH) and in different micellar media. The probe concentration was about 5 μM, and that of the surfactants in all cases was 20 mM. The excitation wavelength employed in all cases was 465 nm.

Figure 2 shows the absorption and corrected emission spectra of C6 in ethanol (EtOH) and micellar solutions of β-C_12_G_2_, TX100 and C_12_E_6_. Table 1 lists the absorption and emission maxima calculated from these spectra. C6 exhibits a broad absorption spectrum with a band maximum at 457 nm in EtOH, which is red shifted around 465 nm in the case of the micellar solutions, indicating the incorporation of the dye in the micelles. A similar effect is seen for the steady-state emission spectra, where the wavelength emission maxima shift from 501 nm in EtOH to around 506 nm in the micellar media. In other words, although the absorption and emission maxima are moderately changed in relation to EtOH, they prove slightly sensitive to the nature of the micelles, suggesting that the microenvironment around C6 is similar in all of the micellar media. Taking into account the data from the literature on the spectroscopic properties of C6 in polar and nonpolar solvents [30], we conclude that the dye is located in the micellar hydration layer, where it senses a relatively polar environment. In addition, the quantum yield of fluorescence (Φ*_f_*) of C6 also remains unchanged (see Table 1).

**Table 1:** Absorption, (λ_abs_)_max_, and emission maxima, (λ_em_)_max_, quantum yields, Φ*_f_*, fluorescence lifetimes, τ*_f_*, and the radiative (*k_r_*) and nonradiative (*k_nr_*) decay rate constants of Coumarin 6 (C6) in ethanol (EtOH) and in different micellar media at 25 °C.

Medium	(λ_abs_)_max_ (nm)	(λ_em_)_max_ (nm)	Φ*_f_*	τ*_f_* ^a^ (ns)	*k_r_* (ns^−1^)	*k_nr_* (ns^−1^)
EtOH	457.0	501.0	0.78	2.57(1.08)	0.304	0.085
β-C_12_G_2_	465.0	505.5	0.77	3.21(1.07)	0.240	0.072
TX100	465.0	507.5	0.78	2.90(1.03)	0.269	0.076
C_12_E_6_	464.5	507.0	0.78	2.80(1.15)	0.279	0.078

^a^ The error limit in τ*_f_* values is ±0.01 ns. Within parentheses are the reduced chi-square (χ^2^) values.

**Figure 3:**
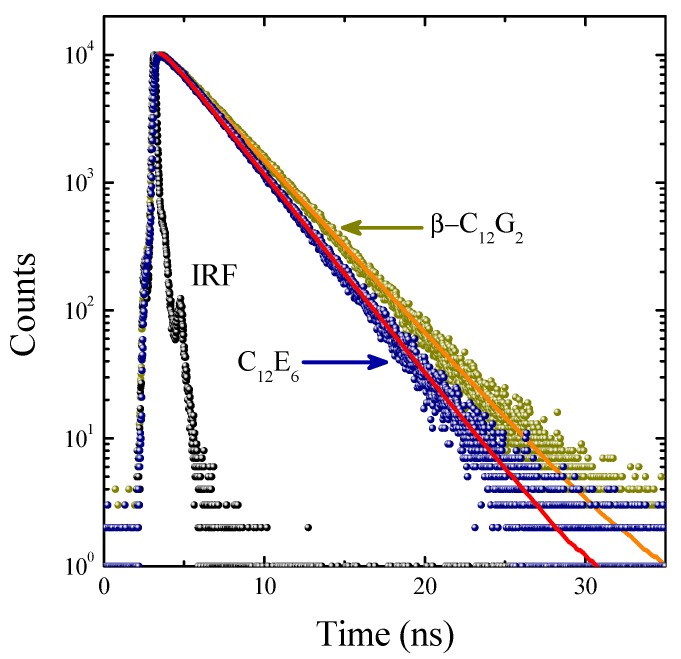
Fluorescence decay curves of C6 together with the instrumental response function (IRF) in micelles of β-C_12_G_2_ and C_12_E_6_ at 25 °C (λ_exc_ = 405 nm and λ_em_ = 505 nm). The solid lines through the data points represent the best fits to single-exponential functions.

Because the excited-state lifetime (τ*_f_*) is a sensitive parameter in probing the local environment of dyes, the fluorescence decay profiles of C6 were recorded by using a time-correlated single-photon counting (TCSPC) technique. Figure 3 shows representative decay curves of C6 in the micellar media of β-C_12_G_2_ and C_12_E_6_. Invariably, C6 exhibited single-exponential decays with lifetime values ranging from 2.57 ns in EtOH to 3.21 ns in β-C_12_G_2_ micelles, as listed in Table 1. These observations are consistent with previously-reported data in similar nonionic micellar media [24].

In addition, it is important to mention that it has previously been established that the nature of the single exponential function of the decay curves of a probe solubilized in different micellar assemblies can be taken as evidence that the probe is located mostly at similar sites of different micellar systems [36].

The data in Table 1 indicate that the lifetime of C6 in micellar media was higher than in EtOH, probably due to the suppression of several non-radiative channels in the micelles and protection from specific solute-solvent hydrogen bonding interactions. Furthermore, in Table 1, the lifetime of C6 slightly decreases from 3.21 ns in β-C_12_G_2_ to 2.90 ns in TX100 and 2.80 ns in C_12_E_6_ micelles, indicating that the dye underwent a marginally higher polarity for the microenvironment in the two last micellar systems. The observed reduction in τ*_f_* values suggests a higher degree of hydration in the palisade layer of the ethoxylated surfactant micelles.

To gain additional insight into the changes of the excited-state photophysical properties of C6 within the micelles studied, we determined the radiative (*k_r_*) and non-radiative (*k_nr_*) decay rate constants for C6 by using [37]:

(1) $$k_{r}=\frac{\Phi_{f}}{\tau_{f}}$$

(2) $$k_{nr}=\frac{1}{\tau_{f}}-k_{r}$$

Rate-constant values found in EtOH and micellar media are also tabulated in Table 1. These data indicate that *k_r_* and *k_nr_* show the same behavior. First, these parameters decrease in the micellar media as compared to EtOH, probably indicating that the polarity sensed by the dye in the confined media is slightly higher than in EtOH, but in the micellar media, some non-radiative channels appear to be suppressed in relation to the homogeneous medium of EtOH. Specifically, the reduction in *k_nr_* in micellar media is the result of two effects: (1) the probe molecule located in the micellar palisade layer experiences a rigid and confined microenvironment where the solvent mediated non-radiative pathway is reduced; and (2) micelles provide protection from specific solute-solvent hydrogen bonding interactions, producing the suppression of some non-radiative channels. Second, in the micellar media, both parameters increase from the maltoside to the ethoxylated surfactants, reaching the maximum values in C_12_E_6_ micelles. This result reflects, on the one hand, the higher polarity of the solubilization site of C6 in the ethoxylated micelles and, on the other, lower micellar microviscosity as compared to that of β-C_12_G_2_, due to the bulkier head groups present in these micelles.

### 2.2. Temperature-Dependent Studies

It is well known that the solution behavior of APG surfactants substantially differs from that of the ethoxylated ones [17,18,19]. For comparison, we have listed in Table S1, in the Supplementary Materials section, the literature values of the critical micelle concentration (CMC) of the three micellar systems studied at different temperatures. In that section, we also provide the aggregation number data previously reported as a function of temperature. In particular, the temperature dependence of the solution properties of APG surfactants is much less pronounced, not showing the typical clouding phenomenon of the ethoxylated surfactants [26,28]. In fact, many physicochemical properties of APG surfactants are almost insensitive to temperature changes, this being attributed mainly to the strength of the hydrogen bonds between the hydroxyl groups of APG surfactants and water and, hence, the head-group dehydration when the temperature increase is much less significant [19].

In this context, to gain additional comparative information on our micellar systems, we performed steady-state fluorescence anisotropy and lifetime measurements of C6 in micellar media, together with dynamic light scattering (DLS) measurements of micelles under temperature changes.

#### 2.2.1. Steady-State Fluorescence Anisotropy

**Figure 4:**
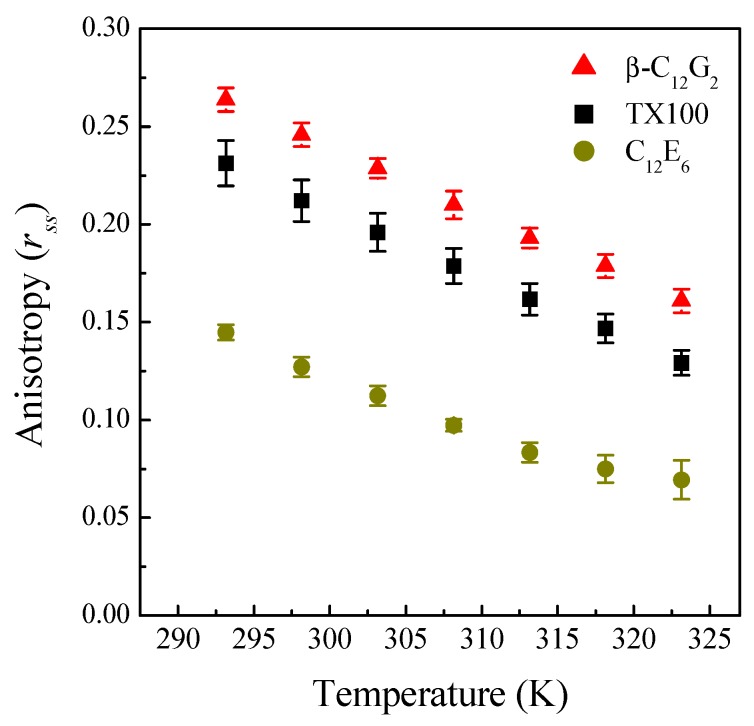
Steady-state fluorescence anisotropy, *r_ss_*, of C6 micellar solutions as a function of temperature.

Steady-state fluorescence anisotropy, *r_ss_*, is related to the viscosity around the probe, η, by Perrin’s equation [37]:

(3) $$\frac{r_{0}}{r_{ss}}=1+\frac{k_{\text{B}}\;T\;\tau_{f}}{V\;\eta }$$

where *r*_0_ is the fundamental anisotropy, *k*_B_ is the Boltzmann constant, *T* is the absolute temperature and *V* and τ*_f_* are the effective volume and the fluorescence lifetime of the probe, respectively. In other words, larger anisotropy values would correspond, *a priori*, to a more rigid environment around the probe at a fixed temperature. Figure 4 shows the *r_ss_* values that we determined as a function of temperature for each micellar system, where it can be seen that, in all of the cases, the anisotropy of C6 decreases monotonically with increasing temperature.

Moreover, the data in Figure 4 could be interpreted in the sense that the micellar microenvironment around C6 becomes less rigid at each temperature in the following order: β-C_12_G_2_ > TX100 > C_12_E_6_. However, steady-state fluorescence data in micelles must be carefully analyzed, because they can be affected by several factors. According to Equation (3), anisotropy values clearly depend on the fluorescence lifetime of the probe, τ*_f_*, and therefore, it is crucial to gather information on the trend of τ*_f_* under the experimental conditions used in the polarization assays. On the other hand, it is well known that the fluorescence depolarization of a probe in micelles is usually attributed to two different rotational processes [38]: (1) rotational diffusion of the probe into the micelle; and (2) rotation of the micelle itself. This second rotational process depends on the micellar size, and thus, the influence of temperature on the micellar size of our systems must be also studied.

#### 2.2.2. Time-Resolved Fluorescence

**Figure 5:**
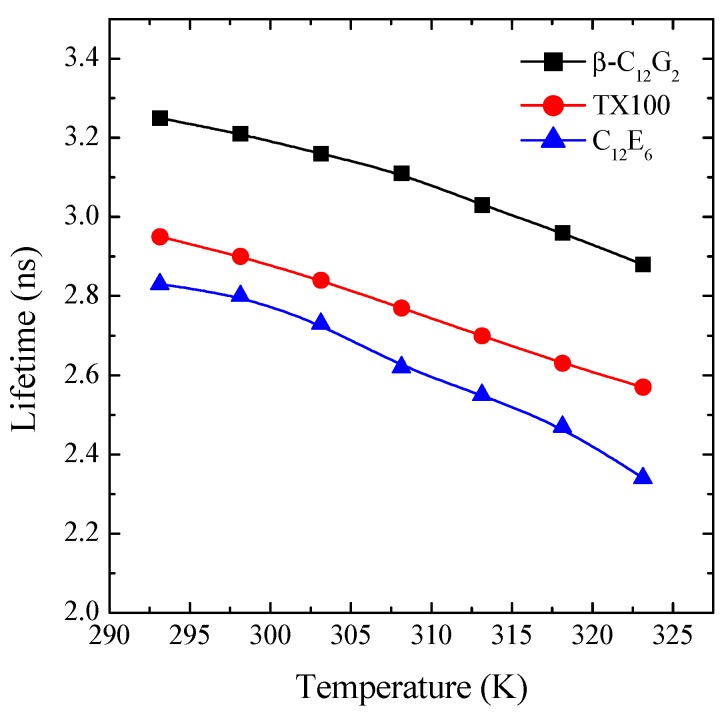
Effect of temperature and the nature of the micellar system on the lifetime values of C6 solubilized in the micellar media (λ_exc_ = 405 nm and λ_em_ = 505 nm).

The time-resolved fluorescence decay of C6 in micellar solutions of the three different non-ionic surfactants were recorded in the temperature range from 293.15–323.15 K. It was found that, in all of the cases, the decay curves were well fitted by a single exponential function. Note that this observation is consistent with a probe fully micellized, that is located at only one site in the micelle [36,39,40,41]. The effect of temperature and the micellar system on the lifetime of C6 is shown in Figure 5. This figure shows a similar behavior in all of the systems, *i.e.*, a reduction of τ*_f_* with temperature, this becoming slightly more pronounced for C_12_E_6_. Our data indicate that within the temperature range from 293.15–323.15 K, the lifetime values of C6 decrease by around 11% for β-C_12_G_2_, 13% for TX100 and 17% for C_12_C_6_.

At a fixed temperature, a reduction in the lifetime values can be correlated with a higher degree of hydration in the micellar palisade layer of the ethoxylated surfactants compared to β-C_12_G_2_. Because ethoxylated surfactants have flexible and polymer-like head groups, their micellar palisade layers become less compact, allowing higher water penetration. Note that this mechanism is consistent with a less rigid microenvironment, which would be reflected in lower values of fluorescence anisotropy, as observed from steady-state fluorescence anisotropy data (see Figure 4).

On the other hand, the more pronounced reduction in τ*_f_* with temperature for the ethoxylated surfactants is indicative of higher alterations in the micellar structure of these surfactants as a result of a higher temperature. Probably, this trend is closely related to the different hydration mechanism of APG surfactants as compared to ethylene oxide-based ones [24], an aspect that will be more extensively discussed in the next section.

#### 2.2.3. Dynamic Light Scattering

**Figure 6:**
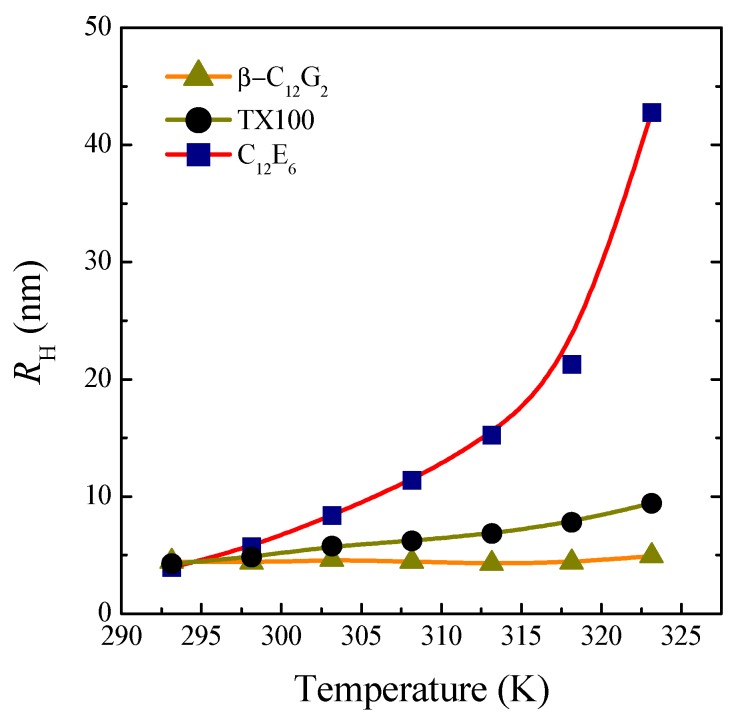
Apparent hydrodynamic radius of micelles, *R*_H_, as a function of temperature.

Figure 6 shows the apparent hydrodynamic radius, *R*_H_, as a function of temperature for the three surfactants studied. It can be seen that, whereas the size of β-C_12_G_2_ remains almost constant when the temperature is raised, the ethoxylated surfactants show a growth that becomes dramatic for C_12_E_6_. This different behavior of sugar-based surfactants compared to the ethoxylated ones has previously been reported [24,25,26,27,28] and is attributed to the different hydration mechanism between the two types of surfactants. Specifically, the stronger H-bonds between the sugar head and surrounding water molecules as compared to ethoxylated surfactants explains the fact that the micellar hydration interacting with the head groups of β-C_12_G_2_ is practically unaffected by temperature. It bears mentioning at this point that sugar-based surfactants do not present clouding, a typical phase-separation process, characteristic of the ethoxylated surfactants, which is attributed to the decrease in the intermicellar repulsions resulting from the reduced hydration of the oxyethylene hydrophilic groups with a rise in temperature [42].

With regard to the different behavior between the two ethoxylated surfactants, it should be noted that the higher cloud-point temperature of TX100 (≈76 °C) as compared to that of C_12_E_6_ (≈52 °C) is due to its shorter hydrocarbon chain length, causing a stronger solvophilicity and, hence, a higher cloud-point temperature [42].

Finally, it is to be noted that the thermal stability observed for β-C_12_G_2_ within the temperature range investigated can become advantageous in surfactant-based applications where the maintenance of the structural properties with temperature are required.

### 2.3. Time-Resolved Anisotropy Studies

**Figure 7:**
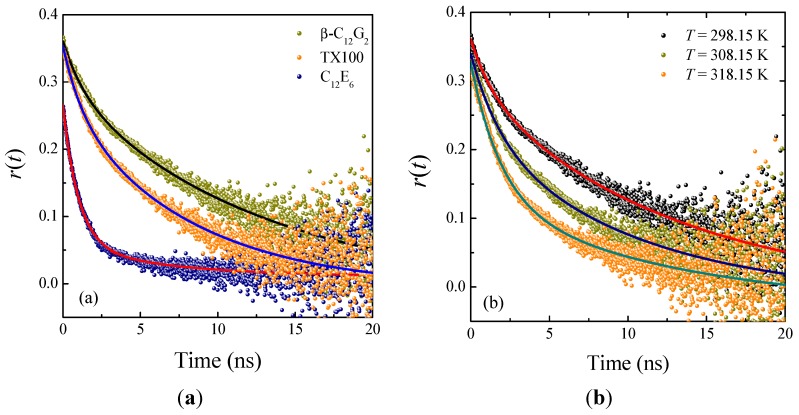
Time-resolved fluorescence anisotropy decays of C6 in: (**a**) micellar media of β-C_12_G_2_, TX100 and C_12_E_6_ at 25 °C; and (**b**) micellar media of β-C_12_G_2_ at different temperatures. The solid lines through the data points are the best fit to Equation (4).

To gain better insight into the effect of temperature on the microenvironmental properties of our nonionic micellar systems, we measured the time-resolved fluorescence anisotropy of C6 in micelles at different temperatures. This technique is a sensitive indicator of the rotational-relaxation of a molecular probe in organized assembly [37]. Typical anisotropy decay profiles of C6 in the micellar systems studied are displayed in Figure 7. These decays were found to be biexponential in nature, which is attributed mainly to the occurrence of various kinds of rotational motions, rather than to different locations of the probe in the micelle [43]. Anisotropy decays of probes solubilized in micelles are often analyzed by the so-called two-step model [44,45]. According to this approach, the rotational motion of the probe into the micelle is characterized by two time constants, as described by [43]:

(4) $$r\left(t\right)=r_{0}\left[\beta \exp\left(-\frac{t}{\tau_{\text{slow}}}\right)+\left(1-\beta \right)\exp\left(-\frac{t}{\tau_{\text{fast}}}\right)\right]$$

in which *r*_0_ is the anisotropy at time zero, τ_slow_ and τ_fast_ are the two reorientation times of the probe in the micelle and β is a pre-exponential factor giving the relative contributions of both time constants. According to the two-step model, the probe undergoes two different movements, *i.e.*, a slow lateral diffusion at or near the micellar interface and a fast wobbling motion in the micelle, with both of these motions being coupled to the rotation of the micelle as a whole [33,44].

The average reorientational times, ‹τ*_r_*›, for all of the systems studied were calculated using the equation:

(5) $$\langle \tau_{r}\rangle =\beta \;\tau_{\text{slow}}+\left(1-\beta \right)\;\tau_{\text{fast}}$$

Table 2 lists the rotational-relaxation parameters found upon fitting our decay curves according to Equation (4). It should be noted that the results were neither reproducible nor reliable in the case of C_12_E_6_ at high temperatures. This was probably due to some type of relaxation process occurring on a timescale shorter than the instrument response, In other words, the kinetics of the probe in C_12_E_6_ micelles appears to be too rapid to be entirely resolved by our single-photon-counting experimental setup. Therefore, from here on, we limit the temperature-dependent study to the cases of β-C_12_G_2_ and TX100.

**Table 2:** Rotational-relaxation parameters for C6 in nonionic micellar systems at different temperatures.

Micelles	*T* (K)	*r*_0_	β ^a^	τ_slow_ (ns)	τ_fast_ (ns)	χ^2^	‹*τ_r_*› (ns)
	298.15	0.373	0.82	12.7 ± 0.8	1.4 ± 0.1	1.02	10.7 ± 3.0
β-C_12_G_2_	308.15	0.355	0.69	10.4 ± 1.0	1.8 ± 0.1	1.09	7.7 ± 1.8
	318.15	0.359	0.45	13.0 ± 2.8	1.9 ± 0.1	1.10	6.9 ± 2.3
	298.15	0.361	0.76	8.1 ± 0.4	1.2 ± 0.1	1.01	6.4 ± 1.6
TX100	308.15	0.359	0.55	7.2 ± 0.8	1.4 ± 0.1	1.21	4.5 ± 1.4
	318.15	0.338	0.25	7.7 ± 1.4	1.4 ± 0.1	1.18	3.0 ± 1.0
C_12_E_6_	298.15	0.306	0.16	10.8 ± 3.4	1.1 ± 0.1	1.21	2.7 ± 1.3

^a^ Uncertainty limits, ∆β, are ≤0.02.

From the data in Table 2, it is first seen that the *r*_0_ values differ among themselves and from that obtained from steady-state anisotropy measurements of C6 in media of high rigidity (*r*_0_ = 0.366) [31]. However, the *r*_0_ values obtained as a fitting parameter of Equation (4) are not reliable, because they depend strongly on the selected left limit of the fitting range, as observed in earlier studies [46]. Therefore, the *r*_0_ values so obtained must be considered not as the limiting anisotropy, but as the anisotropy at time zero. Furthermore, we find that the rotational-relaxation time, given by the average reorientational time of the probe in the micellar environment of the ethoxylated surfactants at 298.15 K, is faster than the corresponding value for β-C_12_G_2_, indicating a considerably lower degree of rigidity in those micellar environments, corroborating our previous observations from steady-state anisotropy data. The data in Table 2 also indicate that when the temperature is raised, the rotational-relaxation becomes faster in both β-C_12_G_2_ and TX100. However, the average reorientational time underwent a higher relative reduction in TX100. Note that this finding is consistent with a more extended dehydration in the ethoxylated surfactant, as discussed above.

On the other hand, a further aspect deserves comment. The data in Table 2 indicate that the average rotational time of C6 in TX100 at 298.15 K is considerably greater than the corresponding value for C_12_E_6_, suggesting a more rigid microenvironment for TX100. A similar result has been recently reported by Pal *et al.* [47] in the case of Coumarin 500 solubilized in micelles of sodium dodecyl sulfate (SDS) and sodium dodecylbenzene-sulfonate (SDBS). These authors attributed this finding to the so-called π-stacking effect, brought about by the attractive noncovalent interaction between aromatic rings.

Another important parameter accounting for the motional restriction of the probe in the micelle is the so-called generalized order parameter, *S*, which is related by the pre-exponential factor β by the relationship *S^2^ =* β [44]. The order parameter provides a measurement of the equilibrium orientational distribution of the probe in the micelle, and their values range from zero, for unrestricted motion, to one for completely restricted motion. Focusing on β values found for β-C_12_G_2_ and TX100, we find that the corresponding *S* values range between 0.90 and 0.50, which are considered high values for this parameter, and they can be taken as an indication that the probe is located in the micellar palisade layer, closer to the interface, rather than the interior of the micelles, where there is a lower degree of order [33].

Now, to gain additional insight into the effect of temperature on the microenvironmental properties of our micellar systems, we tried to estimate the microviscosity of micelles. In this respect, we assumed that the average rotational times of C6 in micelles followed the Stokes–Einstein–Debye (SED) hydrodynamic model of rotational diffusion. According to this approach, the ‹τ*_r_*› values of a non-interacting probe in a medium of local viscosity (or microviscosity), η_m_, is given by:

(6) $$\langle \tau_{r}\rangle =\frac{V_{\text{H}}\;\eta_{\text{m}}}{k_{\text{B}}\;T}$$

where *V*_H_ is the hydrodynamic volume of the probe and *k*_B_ and *T* are the Boltzmann constant and absolute temperature, respectively. As mentioned above, the experimentally-determined values of the average rotational times can result from two different contributions: the rotation of the probe in the micelle and the rotation of the micelle itself [33,38]. The latter rotational motion proves more significant in the case of small micelles, *i.e.*, when its reorientational time is comparable to the average rotational time of the probe, and therefore, we need to evaluate the effect of the micelle size on this parameter. To do so, we use the following relationship [33]:

(7) $$\frac{1}{\langle \tau_{r}\rangle }=\frac{1}{{\langle \tau_{r}\rangle }_{\text{P}}}+\frac{1}{\tau_{\text{M}}}$$

where ‹τ*_r_*›_P_ is, properly speaking, the average reorientation time of the probe in the micelle and τ_M_ is the time constant for the overall rotation of the micelle, which can also be evaluated, according to the SED hydrodynamic theory, using the equation:

(8) $$\tau_{\text{M}}=\frac{V_{\text{M}}\;\eta_{\text{sol}}}{k_{\text{B}}\;T}$$

where *V*_M_ is the hydrodynamic volume of the micelle, which can be estimated form the corresponding hydrodynamic radius as determined by DLS measurements, and η_sol_ is the viscosity of the bulk phase.

Moreover, we need to evaluate the hydrodynamic volume of the probe (*V*_H_), which is given by *V_H_ = VfC*_slip_, *i.e.*, the product of the van der Waals volume (*V*), shape factor (*f*) and the boundary condition parameter (*C*_slip_) [31,33]. Consequently, the microviscosity of micelles can be evaluated as:

(9) $$\eta_{\text{m}}=\frac{{\langle \tau_{r}\rangle }_{\text{P}}k_{B}\;T\;}{V\;f\;C_{\text{slip}}}$$

Assuming the values from the literature for C6 [31,33] of 303 Å^3^, 3.13 and 0.46 for *V*, *f* and *C*_slip_, respectively, we can estimate the microviscosity values. Table 3 lists all of these parameters determined in the case of the micellar systems of β-C_12_G_2_ and TX100 at different temperatures. First of all, the data in Table 3 indicate that the rotational-relaxation time of the micelles is remarkably higher than the corresponding average reorientation time of the probe in the micelle, leading us to conclude that the former contributes little to the observed rotational-relaxation dynamics of the probe. With respect to the microviscosity values (η_m_) determined, it is important to remark that they are higher than those determined by Dutt for ionic micelles using C6 as a probe [33], suggesting a tighter microenvironment for nonionic surfactants.

**Table 3:** The effect of temperature on the micellar microviscosity as determined from the average rotational time of C6 in micelles.

Micelles	*T* (K)	‹τ*_r_*› (ns)	‹τ*_r_*› (ns)	τ_M_ (ns)	η_m_ (mPa·s)
β-C_12_G_2_	298.15	10.63	12.41	74.12	117.1
	308.15	7.69	8.78	62.05	85.6
	318.15	6.88	8.03	48.09	80.8
TX100	298.15	6.43	6.86	102.80	64.7
	308.15	4.53	4.65	170.59	45.4
	318.15	2.98	3.01	270.68	30.4

In addition, the microviscosity values of β-C_12_G_2_ are also much higher than those of TX100 at a fixed temperature, reflecting that the bulkier and more rigid head group of the APG surfactant provides a more structured palisade layer as compared to TX100. Moreover, the effect of an increased temperature is visibly more pronounced in the case of the ethoxylated surfactant. Note that when the temperature rises from 298.15–318.15 K, the microviscosity of TX100 micelles is reduced by about 53%, whereas in the case of β-C_12_G_2_, the reduction is about 31%. This result is closely related to a more extended dehydration of the micellar palisade layer of TX100 as the temperature rises, in good agreement with previous observations in this work.

## 3. Experimental Section

### 3.1. Materials

Table 4 lists the provenance and the purity grade of the surfactant samples used in the present study. Due to their high purity grade, all of these substances were used as received. Aqueous stock solutions of surfactants were prepared by weight. Working samples with a lower concentration were prepared daily from the stock solutions. In order to ensure the fully-micellized state of the probe, a surfactant concentration well above the CMC (20 mM) was used in all of the working samples. The ultrapure water (resistivity ~18 MΩ·cm) used to prepare all of the solutions was obtained by passing pure water from a Millipore Elix system through an ultra-high quality Millipore Synergy purification system. The fluorescence probe 3-(2-benzothiazolyl)-7-(diethylamino)-coumarin or Coumarin 6 (C6) was acquired from Exciton (Dayton, OH, USA) (laser grade) and used as received. A 1-mM stock solution of C6 was prepared by weight in absolute ethanol. Measurement samples of about 5 µM in C6 were prepared by adding small volumes of the ethanolic solutions to each micellar solution.

**Table 4:** Surfactants used in the present study.

Chemical Name	Abbreviation	Source	Grade	Mass Fraction Purity
*n*-dodecyl-β-d-maltoside	β-C_12_G_2_	Calbiochem	Ultrol	≥0.99
*p-tert-*octyl-Phenoxy polyethylene (9.5) ether	TX100	Sigma-Aldrich	BioXtra	≥0.98
*n*-dodecyl-hexaoxyethylene-glycol	C_12_E_6_	Sigma-Aldrich	BioXtra	≥0.98

### 3.2. Methods

#### 3.2.1. Steady-State Spectroscopic Measurements

Absorption and emission spectra of ethanolic and micellar solutions of C6 were recorded with a Cecil 2021 UV-VIS spectrometer and a FluoroMax-4 (Horiba Jobin Yvon, Longjumeau, France) spectrofluorometer, respectively. In all cases, a 1-cm path-length quartz cuvette was used. All emission spectra were corrected for the wavelength-dependent response of the detection system, the sample chamber temperature being controlled by a built-in Peltier unit. The steady-state fluorescence anisotropy values, *r_ss_*, were measured in the same apparatus provided with a polarization accessory, which uses the L-format instrumental configuration [37] and an automatic interchangeable wheel with Glan-Thompson polarizers. The anisotropy values were averaged over an integration time of 10 s, and at least three measurements were made per sample.

The fluorescence quantum yields of C6 in the micellar media, Φ*_f_*, were determined using the fluorescence quantum yield of the same probe in ethanol (Φ*_r_* = 0.78) [48] as in the reference and by using the equation:

(10) $$\Phi_{f}=\Phi_{r}\frac{F_{f}\;A_{r}\;n_{f}^{2}}{F_{r}\;A_{f}\;n_{r}^{2}}$$

where *F* is the area under the corrected emission spectrum, *A* is the absorbance at the excitation wavelength and *n* is the refractive index of the solvent used. Subscripts *r* and *f* refer to the reference and to the sample, respectively.

#### 3.2.2. Time-Resolved Measurements

Time-resolved fluorescence measurements were made on a LifeSpec II luminescence spectrometer (Edinburgh Instruments, Ltd., Livingston, UK) based on the time-correlated single-photon counting (TCSPC) technique, using a picosecond pulsed diode laser at 405 nm (Edinburgh Instruments, Ltd., Livingston, UK) at a repetition rate of 20 MHz as the excitation source. The emission was recorded at 505 nm while keeping the emission polarizer at the magic angle of 54.7° with respect to the vertically-polarized excitation beam. To optimize the signal-to-noise ratio, 10^4^ photon counts were collected in the peak channel. The instrumental response function (IRF) was regularly determined by measuring the scattering of a Ludox solution. For this setup, the IRF was about 250 ps at full width at half maximum (fwhm). The decay parameters were determined by reconvolution, and the decay curves were fitted with the help of the FAST software package from Edinburgh Instruments. The intensity decay curves for all lifetime measurements were fitted as a sum of exponential terms:

(11) $$I\left(t\right)=\sum_{i}A_{i}\exp\left(\frac{t}{\tau_{i}}\right)$$

where *A_i_* is a pre-exponential factor of the component *i* with a lifetime τ*_i_*. In these experiments, the temperature was maintained at the desired value using a Peltier system with an accuracy of ±0.1 °C.

Time-resolved fluorescence anisotropy measurements were performed with the same apparatus, which was fitted with an automatic set of polarizers. These experiments were carried out by measuring the fluorescence decays for parallel, *I_VV_*(*t*), and perpendicular, *I_VH_*(*t*), polarizations with respect to the vertically-polarized excitation light. The anisotropy decay, *r*(*t*), was calculated using the relation [37]:

(12) $$r\left(t\right)=\frac{I_{VV}\left(t\right)-G\;I_{VH}\left(t\right)}{I_{VV}\left(t\right)+2G\;I_{VH}\left(t\right)}$$

where the *G* factor was also experimentally determined using a solution of C6 in ethanol, thus ensuring a very fast rotational relaxation of the probe. In all cases, a double-exponential decay was required for the best fit of the anisotropy decays.

In the analysis of either fluorescence or anisotropy decays, the quality of the fits was evaluated by the reduced χ^2^ values and the distribution of the weighted residuals among the data channels. The statistical criteria determining the level of fit were a reduced χ^2^ value of <1.2 and a random distribution of weighted residuals.

#### 3.2.3. Dynamic Light-Scattering Measurements

Micelle size dependence on temperature was characterized by dynamic light scattering (DLS) measurements, which were performed on a Zetasizer Nano-S system (Malvern Instruments, Malvern, UK). This instrument uses a backscattering detection system (scattering angle θ = 173°) and is fitted with a helium-neon laser source (632.8 nm and 4.0 mW) and a Peltier thermoelectric device. The apparent hydrodynamic radii of the micelles, *R_H_*, were calculated from the DLS diffusion coefficients assuming the Stokes–Einstein equation, which related the translational diffusion coefficients, *D*_0_, with *R_H_* by the relationship [49]:

(13) $$D_{0}=\frac{k_{\text{B}}\;T}{6\;\pi \;\eta \;R_{H}}$$

where *k*_B_ is the Boltzmann constant, *T* the absolute temperature and η the solvent viscosity. Micellar solutions of varying composition and fixed concentration (20 mM) were filtered directly into cuvettes through membrane filters (pore size 0.1 μm). The cuvette was rinsed several times with ultrapure water prior to each measurement and then filled with filtered micellar solutions. The DLS data were analyzed using the CONTIN algorithm [50].

## 4. Conclusions

Coumarin 6 was solubilized in three nonionic micellar media that were selected for the purpose of hydrophobic drug solubilization. Steady-state and time-resolved fluorescence studies of C6 in each micellar medium were performed over a range of temperatures from 293.15–323.15 K in order to reflect the solubilization behavior as a function of temperature. Spectroscopic data show that C6 resides in the palisade layer of micelles, where it senses a relatively polar environment. DLS measurements provided insight into the micellar size of the three systems *vs.* temperature. The micelles formed by the APG surfactant remained almost constant in the temperature range considered, while those of the ethoxylated surfactants grew with temperature, this growth being more pronounced in the case of C_12_E_6_. It was established that, although micelles constituted by ethoxylated surfactants are more extensively hydrated, this hydration is more easily reduced by a higher temperature as compared to the APG surfactant, which was attributed to the fact that hydration water is more strongly bound to the sugar head groups than that to those of ethoxylated ones.

In the present work, we have demonstrated that both the structure and the microstructure of the micelles formed by the APG surfactant are more thermally stable compared to those of the ethoxylated ones. Because nonionic surfactants are frequently used as solubilizing agents of hydrophobic molecules in several pharmacological applications, our study shows that APG surfactants can be advantageously employed as an alternative to conventional ethoxylated surfactants, particularly in the cases in which the thermal stability of the system is required.

## Supplementary Materials

Supplementary materials can be accessed at: http://www.mdpi.com/1420-3049/20/10/19343/s1.
