# Supplementary material for: Analysis of the Photophysical Behavior and Rotational-Relaxation Dynamics of Coumarin 6 in Nonionic Micellar Environments: The Effect of Temperature
Source: Molecules. 2015 Oct 23;20(10):19343–60. doi: 10.3390/molecules201019343 (PMC6332106; doi:10.3390/molecules201019343)
Supplement: Supplementary file 1 [file molecules-20-19343-s001.pdf]

Supplementary Materials

Analysis of the Photophysical Behavior and Rotational-Relaxation Dynamics of Coumarin 6 in Nonionic Micellar Environments: The effect of Temperature

Cristóbal Carnero Ruiz, José Manuel Hierrezuelo, José Antonio Molina-Bolivar

Department of Applied Physics II, Engineering School, University of Malaga, Malaga 29071

Micellar properties as a function of temperature

Critical Micelle Concentration (CMC)

Table S1. Literature values of the critical micelle concentration (CMC) of the micellar systems at different temperatures.

T (K)	CMC (mM)		
	**β-$C_{12}G_2$**	**TX100**	**$C_{12}E_6$**
298.15	0.180 [1] 0.150 [2]	0.238 [3] 0.240 [4]	0.073[2] 0.072 [5]
303.15		0.231 [3]	0.068 [5]
308.15		0.221 [4]	
313.15	0.242 [1]		0.063 [5]
318.15		0.217 [4]	
323.15	0.255 [1]		0.060 [5]

Aggregation Number (N_{agg})

Aoudia and Zana have studied the mean aggregation number of β-$C_{12}G_2$ in a range of temperature. They determined that the aggregation number is nearly invariant in the *T*-range 16–60 °C, with an average value of 125 ± 10 [1], which is within the range reported by Baverbäck *et al.* of 137–113 at 25 °C [6].

The effect of temperature on the size of ethoxylated surfactants, including TX100 and $C_{12}E_6$ has been widely reported in the literature. See, for example, references [7−14]. The conclusion attained in these studies is that TX100 shows a modest increase in the aggregation number with temperature in comparison with $C_{12}E_6$. Specifically, it was observed that N_{agg} of TX100 increases with *T*, slowly in the *T*-range 25–40 °C (from 105 to ~150), but much more rapidly above 40 °C. On the contrary, N_{agg} of $C_{12}E_6$ increases dramatically as the temperature undergoes a relatively small change from 30 °C. Essentially the same behavior that we have observed by DLS measurements (see, Figure 6).

Plots in Figure S1 shows selected literature values of the aggregation number of TX100 and $C_{12}E_6$ for comparison.

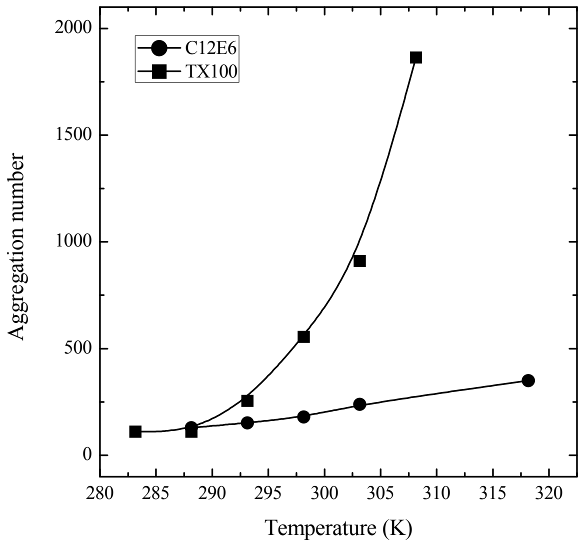

Figure S1. Literature data for the micellar aggregation numbers: (●) TX100 from reference [8] and (■) $C_{12}E_6$ from reference [10], as obtained by sedimentation equilibrium measurements.

References

1. Aoudia, M.; Zana, R. Aggregation behavior of sugar surfactants in aqueous solutions: Effects of temperature and the addition of nonionic polymers. *J. Colloid Interface Sci.* **1998**, *206*, 158–167.
2. Patil, S. R.; Buchavzov, N.; Carey, E.; Stubenrauch, C. Binary mixtures of beta-dodecylmaltoside (β-$C_{12}G_2$) with cationic and non-ionic surfactants: micelle and surface compositions. *Soft Matter* **2008**, *4*, 840–848.
3. Carnero Ruiz, C.; Molina-Bolivar, J.A.; Aguiar, J.; MacIsaac, G.; Moroze, S.; Palepu, R. Thermodynamic and structural studies of Triton X-100 micelles in ethylene glycol-water mixed solvents. *Langmuir* **2001**, *17*, 6831–6840.
4. Ray, A.; Nemethy, G. Micelle formation by nonionic detergents in water-ethylene glycol mixtures. *J. Phys. Chem.* **1971**, *75*, 809–815.
5. Chen, L.-J.; Lin, S.-Y.; Huang, C.-C.; Chen, E.-M. Temperature dependence of critical micelle concentration of polyoxyethylenated non-ionic surfactants. *Colloids Surf. A* **1998**, *135*, 175–181.
6. Bäverback, P.; Oliveira, C.L.P.; Garamus, V.M.; Varga, I.; Claesson, P.M.; Pedersen, J.S. Structural properties of β-dodecylmaltoside and $C_{12}E_6$ mixed micelles. *Langmuir* **2009**, *25*, 7296–7303.
7. Zana, R.; Weill, C. Effect of temperature on the aggregation behaviour of nonionic surfactants in aqueous solutions *J. Physique Lett.* **1985**, *46*, L-953–L-960.
8. Brown, W.; Rymdén, R.; van Stam, J.; Almgrem, M.; Svensk, G. Static and dynamic properties of nonionic amphiphile micelles—Triton X-100 in aqueous solution. *J. Phys. Chem.* **1989**, *93*, 2512−2519.
9. Rau, H.; Greiner, G.; Hammerle, H. Temperature-dependence of number and size of Triton-X-100 micelles in aqueous solution. *Ber. Bunsenges. Phys. Chem.* **1984**, *88*, 116–121.
10. Herrington, T.H.; Sahi, S.S. Temperature-dependence of the micellar aggregation number of *n*-dodecylpolyethyleneoxide surfactants. *J. Colloid Interface Sci.* **1988**, *121*, 107−120.
11. Streletzky, K.; Phillies, G.D.J. Temperature dependence of Triton X-100 micelle size and hydration. *Langmuir* **1995**, *11*, 42−47.
12. Corti, M.; Degiorgio, V. Micellar properties and critical fluctuations in aqueous solutions of non-ionic amphiphiles. *J. Phys. Chem.* **1981**, *85*, 1442−1445.

13. Kato, T.; Seimiya, T. Study on intermicellar interactions and micelle size distribution in aqueous-solutions of nonionic surfactants by measurements of mutual diffusion and self-diffusion coefficients. *J. Phys. Chem.* **1986**, *90*, 3159−3167.
14. Thomas, H.G.; Lomakin, A.; Blankschtein, D.; Benedek, G.B. Growth of mixed nonionic micelles. *Langmuir* **1997**, *13*, 209−218.
